# Signal-to-noise of linear and volume measures of left ventricular and left atrial size

**DOI:** 10.1186/s12947-023-00320-w

**Published:** 2024-01-03

**Authors:** Yunbo Duan, Nezar Amir, Guy P. Armstrong

**Affiliations:** 1 Auckland University School of Medicine, Auckland, New Zealand; 2 North Shore Hospital, Auckland, New Zealand

**Keywords:** Echo volumes, Imprecision, Variability, Accuracy, Serial change

## Abstract

**Background:**

Serial echocardiographic assessments are common in clinical cardiology, e.g., for timing of intervention in mitral and aortic regurgitation. When following patients with serial echocardiograms, each new measurement is a combination of true change and confounding noise.

The current investigation compares linear chamber dimensions with volume estimates of chamber size. The aim is to assess which measure is best for serial echocardiograms, when the ideal parameter will be sensitive to change in chamber size and have minimal spurious variation (noise). We present a method that disentangles true change from noise. Linear regression of chamber size against elapsed time gives a slope, being the ability of the method to detect change. Noise is the scatter of individual points away from the trendline, measured as the standard error of the slope. The higher the signal-to-noise ratio (SNR), the more reliably a parameter will distinguish true change from noise.

**Methods:**

LV and LA parasternal dimensions and apical biplane volumes were obtained from serial clinical echocardiogram reports. Change over time was assessed as the slope of the linear regression line, and noise was assessed as the standard error of the regression slope. Signal-to-noise ratio is the slope divided by its standard error.

**Results:**

The median number of LV studies was 5 (4–11) for LV over a mean duration of 5.9 ± 3.0 years in 561 patients (diastole) and 386 (systole). The median number of LA studies was 5 (4–11) over a mean duration of 5.3 ± 2.0 years in 137 patients.

Linear estimates of LV size had better signal-to-noise than volume estimates (*p* < 0.001 for diastolic and *p* = 0.035 for systolic). For the left atrium, the difference was not significant (*p* = 0.214). This may be due to sample size; the effect size was similar to that for LV systolic size. All three parameters had a numerical value of signal-to-noise that favoured linear dimensions over volumes.

**Conclusion:**

Linear measures of LV size have better signal-to-noise than volume measures. There was no difference in signal-to-noise between linear and volume measures of LA size, although this may be a Type II error. The use of regression lines may be better than relying on single measurements. Linear dimensions may clarify whether changes in volumes are real or spurious.

**Graphical Abstract:**

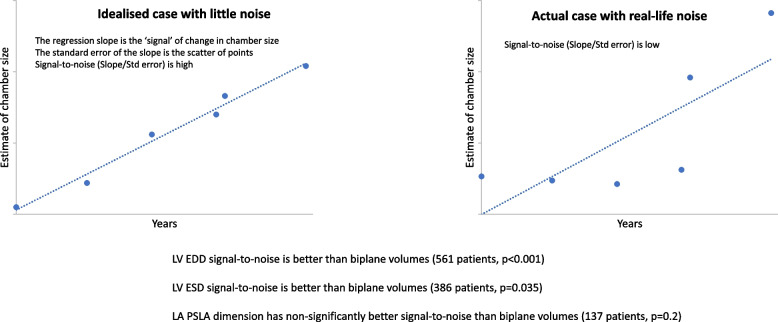

## Background

Serial echocardiographic assessments are common in clinical cardiology, e.g., for timing of intervention in mitral and aortic regurgitation. Unfortunately, each new measurement is a combination of true change and confounding noise.

Current recommendations prioritise the assessment of chamber size with volume rather than linear dimension [1]. Volumes better approximate the geometry of cardiac chambers and facilitate comparison with MRI and CT. Volumes reflect changes in chamber size in more dimensions than a single linear measurement. However, they require multiple measurements from two apical windows with sometimes challenging image quality. The linear dimension only requires a single measurement from an adequate parasternal window.

Serial echocardiograms over days to weeks will vary due to spurious noise only. The time frame is too short for true biological changes in chamber size. Yet even such short-term studies found left ventricular (LV) and left atrial volumes (LA) to be excessively variable [2, 3]. This must be noise, rather than a signal of changing size. However, the clinical need is for precise estimates of true change in chamber size over months to years. This longer-term assessment of volumes requires disentangling true change in chamber size from confounding noise.

The current investigation evaluates linear dimensions and volume estimates of chamber size for their ability to detect change over time. The aim is to assess which measure is best for serial echocardiograms, for which the ideal parameter will be both sensitive to changes in chamber size and have minimal spurious variation (noise).

**Fig. 1:**
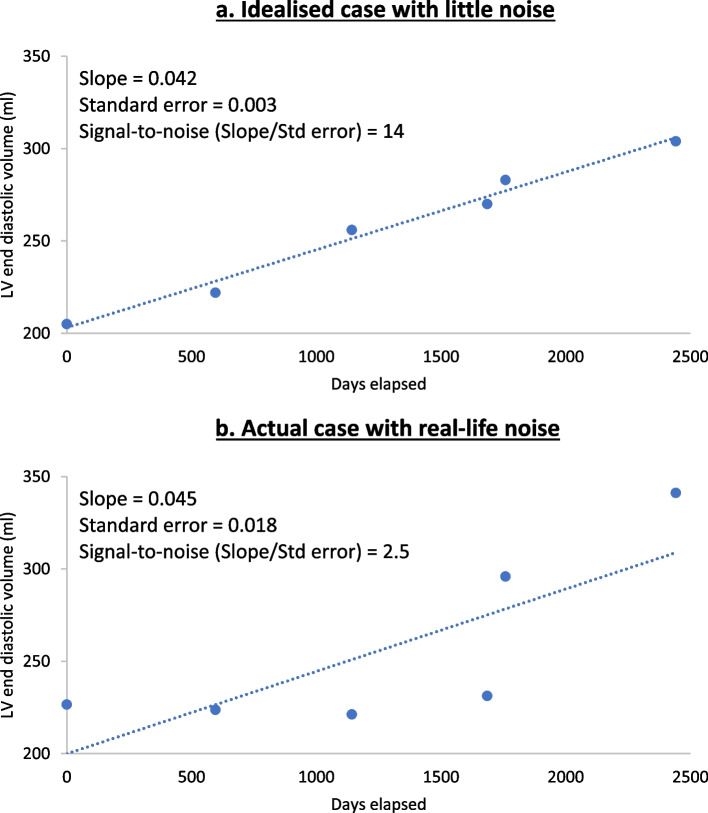
Linear regression technique for calculating signal-to-noise. **a** and **b** are regression plots of LV end-diastolic volume against time. The slope of the line is the best estimate of the true change in volume over time. This is the ‘signal’ of change in size we are seeking. The scatter of points is noise, quantified by the standard error. The signal-to-noise ratio (SNR) is the slope divided by the standard error. As it is dimensionless, signal-to-noise ratio is suitable for comparing variables with different units, such as linear dimensions and volumes. **a** is an idealised case with little scatter or noise. The signal-to-noise ratio is 14, indicating an excellent ability to distinguish true change from noise. **b** is a real case with realistic noise. The signal-to-noise ratio is only 2.5, indicating less reliability in distinguishing true change from noise

The problem can be addressed with linear regression of chamber size against elapsed time, then applying the concept of signal-to-noise, as illustrated in Fig. 1 [4, 5]. Firstly, the preferred parameter will be sensitive to changes in chamber size; the steeper the slope of the regression line, the more sensitive the parameter to change. The regression slope is the best estimate of true change with time; the trend represented by the slope is the ‘signal’ of change being sought. Secondly, the preferred parameter, will have individual data points close to the fitted regression line (as measured by the standard error of the slope). Less scattered points indicate less spurious noise obscuring the true change in chamber volume. The ideal parameter to distinguish true change from spurious noise will have a large slope and a small standard error. Combining them gives the signal-to-noise ratio (SNR), equal to the slope divided by standard error. Being dimensionless, it can compare parameters with different scales, such as linear dimension and volume. For tracking change over time, a high signal-to-noise ratio matters more than an accurate representation of chamber size.

## Methods

From our clinical echo database between 2005 and 2019, reports were extracted for all adult patients with 4 or more serial studies. Cases were included only if there was complete data for linear and biplane volumetric estimates of LV and LA chamber size. Echocardiograms were performed using a variety of fully featured GE and Phillips machines. Sonographer numbers fluctuated and included supervised trainees. To evaluate signal-to-noise in the real-world clinical setting, values for linear chamber dimensions and volumes were taken from the clinical report. No images were reviewed or remeasured. Image acquisition and analysis followed guideline recommendations [1]. Linear measurements were obtained from the parasternal long axis window using M-mode or 2D. Volumes were measured from dedicated apical 2D views using the biplane method of disk [1].

### Statistical analysis

Linear chamber dimensions and volumes were compared using the signal-to-noise ratio for changes over time, defined as the linear regression slope divided by the standard error of the slope. To facilitate linear regression analysis, patients were excluded if they underwent a discrete intervention that could cause a step or fluctuating change in cardiac chamber size. This included heart surgery, heart transplant, biventricular pacing, bariatric surgery, large pericardial effusion and treatable cardiomyopathies, including anticancer agents, sarcoidosis, peripartum and rhythm-related cardiomyopathies.

For each patient, the LV and LA size parameters underwent linear regression against elapsed time (Fig. 1). The slope of this line (B coefficient) represents the change in chamber size over time; this trend is the signal of change being sought. The scatter of individual data points away from the fitted regression line is the noise that obscures the signal; represented by the standard error of the slope (standard error of the B coefficient). Thus, the signal-to-noise ratio is the slope divided by the standard error of the slope. Dimensional analysis shows that the signal-to-noise ratio for linear dimension has units $\frac{Slope\;\left(cm/days\right)}{Standard\;error\;\left(cm/days\right)}$ The units cancel so that signal-to-noise is a dimensionless quantity, and this applies similarly to the volume measurements. This makes signal-to-noise ratio ideal for comparing variables with different units, such as centimetres (chamber dimension) and millitres (chamber volume). For each patient, we calculated the difference in the signal-to-noise ratio between linear and volume measures. A one-sample two-sided Student’s t test was used to determine whether this differed from zero, *p* < 0.05. Linear modelling was chosen as visual inspection of the data was consistent with linearity and the small number of observations for each patient risked curve over-fitting by more complex regression [6]. Logarithmic transformation of the data did not alter the findings.

A sample size of 199 patients has 80% power to detect a 20% difference in signal-to-noise. Data were analysed with Microsoft Excel version 1903 and SPSS version 29.0.0.0.

## Results

The median number of left ventricle (LV) studies was 5 (range 4–11) for LV over a mean duration of 5.9 ± 3.0 years in 561 patients (diastole) and 386 patients (systole) (Table 1). The median number of left atrial studies was 5 (range 4–11) over a mean duration of 5.3 ± 2.0 years in 137 patients. The primary indication for echo was valvular heart disease or aortopathy (50%), cardiomyopathy or myocarditis (27%), ischemic heart disease (14%), chemotherapy (5%), endocarditis (3%), and other (1%).

**Table 1:** Descriptive statistics for the data overall

	n	Linear	Volume
**LV diastole**	561	5.5 ± 0.9 cm	141.2 ± 55.1 ml
**LV systole**	386	4.2 ± 1.0 cm	79.8 ± 45.7 ml
**LA**	137	4.6 ± 0.9 cm	100.6 ± 60.0 ml

**Fig. 2:**
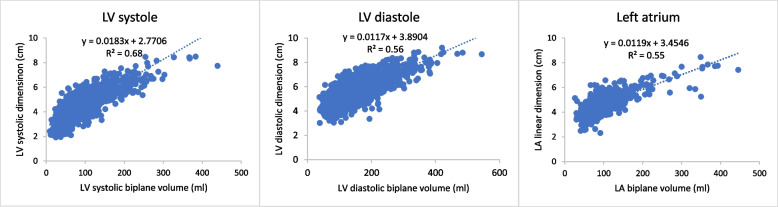
Scatter plot of linear dimensions against volumes. There is a modest correlation between linear and volume measures, similar to other reports [7–10]

Figure 2 is a scatter plot of linear dimensions against volumes.

**Fig. 3:**
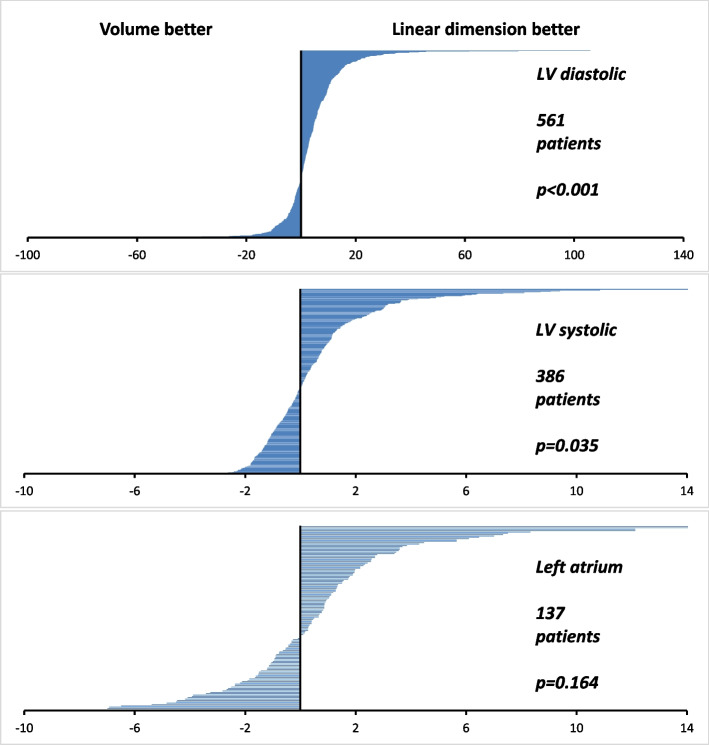
Signal-to-noise difference between linear and volume estimates of cardiac chamber size. Individual patient data. Difference in signal-to-noise between linear and volume estimates of cardiac chamber size. From individual patient’s linear regressions. Signal-to-noise difference is on the horizonal axis

Linear estimates of LV size had better signal-to-noise than volume estimates, *p* < 0.001 for diastolic and *p* = 0.035 for systolic (Table 2 and Fig. 3). For the left atrium, the difference was not significant (*p* = 0.214), although the sample size lacked statistical power. All three parameters had a numerical value of signal-to-noise that favoured linear dimensions over volumes.

**Table 2:** Average values of signal-to-noise difference

	Linear-volume difference	Effect size	*p* value
**LV diastole (*n* = 561)**	3.738 ± 13.168	0.28	< 0.001
**LV systole (*n* = 386)**	0.334 ± 3.152	0.11	0.035
**LA (*n* = 137)**	0.344 ± 3.220	0.12	0.214

Difference between linear and volume values of signal-to-noise ratio. The value is positive when the linear measurement has a better signal-to-noise. Effect size is the Cohen’s d point estimate

## Discussion

When following change over time in the size of the left ventricle, the signal-to-noise ratio is higher for linear dimensions than for volumes. This suggests that linear dimensions more reliably detect true change in LV size. A parameter with a higher signal-to-noise ratio contains relatively more information on true change, and relatively less misleading noise. A similar trend exists for left atrial dimension having higher signal-to-noise than biplane volumes, but the power calculation suggests the sample size was too small to show significance.

The noise (variability) in volumes is consistent with earlier studies [2, 3]. The current study extends the findings to the contemporary era, over longer follow-up and using clinical (not research) echo data. We are not aware of other studies evaluating the diagnostic test performance of serial measurements of echocardiographic chamber size.

Many factors contribute to noise when a parameter is measured repeatedly over time. The typical assessment of reproducibility reported in cross-sectional research does not account for all sources of variability [11]. Variability can stem from the biology, personnel, or measurement process. Serial measurements will vary due to normal alterations in a patient’s volume status, heart rate and blood pressure. Individual sonographers differ in how images are acquired, in the cine loop and frame selected for measurement and in the nuance of how the measurement is conducted. Volume measurements are noisier when endocardial definition is impaired by poor far-field lateral resolution or echo dropout in walls parallel to insonation. It is challenging to acquire non-foreshortened images from the apical two-chamber view. Volume calculations require arithmetic manipulation whereby errors are magnified by multiplication. There are precedents for simple single measurements outperforming more representative variables derived from arithmetic manipulation of multiple measurements. In aortic stenosis with normal LV function, peak jet velocity predicts outcome better than noisier valve area [12].

This study describes a technique to control for the effect of noise when assessing a parameter’s change over time. Applying the principles of signal-to-noise analysis to linear regression, it determines the optimal parameter for assessing temporal change. The signal-to-noise ratio described is suitable for longitudinal studies where there is a need to separate true change from noise. It differs from conventional methods for assessing noise (variability) that are used in cross-sectional and short-term studies. These more familiar methods treat all change as noise. Therefore, they are only appropriate for situations where no true change is expected.

### Limitations

The findings need confirmation from other centres and from larger samples. This study used clinical data, which will be noisier than research data but with better external validity. To enable use of linear regression, we included cases with 4 or more serial studies and excluded cases where an abrupt change in chamber size was possible. However, the principle that linear dimensions more reliably detect serial change applies to all cases, including those excluded from this validation study. This study does not address noise in serial 3D echocardiographic volumes, as this is not part of our routine clinical echo examination.

### Clinical relevance

Echocardiography is commonly used for serial surveillance, e.g., in patients with valvular regurgitation, to determine when LV size meets the threshold for surgery. Linear dimensions are have better signal-to-noise than volume measures and so will more reliably detect true changes in LV size. This study provides a mechanistic explanation for findings that both linear dimensions and volumes can be predictive, independently of each other [9, 13, 14]. Combining the use of linear and volume parameters may be better than using one or the other. Linear dimensions and volumes are obtained from different echo windows using different measurements. Therefore, each may contribute independent information on cardiac chamber size and change. Additionally, plotting a linear regression trendline through serial data points gives a more accurate indication of chamber size than a single point estimate.

## Conclusions

For detection of change over time, linear measures of LV size have better signal-to-noise than volume measures. There was no significant difference in signal-to-noise between linear and volume measures of LA size, although this may be a Type II error. The use of regression lines may be better than relying on single measurements. Linear dimensions may clarify whether changes in volumes are real or spurious.

## Data Availability

The data underlying this article will be shared on reasonable request to the corresponding author.

## Abbreviations

LV: left ventricle
LA: left atrium
LV EDD: left ventricular end diastolic dimension
LV ESD: LV end systolic dimension
MRI: magnetic resonance imaging
CT: computed tomographic imaging
